# Physiological Characteristics of Female Soccer Players and Health and Performance Considerations: A Narrative Review

**DOI:** 10.1007/s40279-021-01458-1

**Published:** 2021-04-12

**Authors:** Rebecca K. Randell, Thomas Clifford, Barry Drust, Samantha L. Moss, Viswanath B. Unnithan, Mark B. A. De Ste Croix, Naomi Datson, Daniel Martin, Hannah Mayho, James M. Carter, Ian Rollo

**Affiliations:** 1 Gatorade Sports Science Institute, Life Sciences R&D, PepsiCo, Leicester, UK; 2 School of Sport, Exercise and Health Sciences, Loughborough University, Loughborough, UK; 3 School of Sport, Exercise and Rehabilitation Sciences, University of Birmingham, Birmingham, UK; 4 Department of Sport and Exercise Sciences, University of Chester, Chester, UK; 5 Institute of Clinical Exercise and Health Science, Division of Sport and Exercise, School of Health and Life Sciences, University of the West of Scotland, Hamilton, Scotland, UK; 6 School of Sport and Exercise, University of Gloucestershire, Gloucester, UK; 7 Institute of Sport, University of Chichester, Chichester, UK; 8 School of Sport and Exercise Science, University of Lincoln, Lincoln, UK; 9 Sports Science Department, Manchester City Football Club, Manchester, UK

## Abstract

Female soccer has seen a substantial rise in participation, as well as increased financial support from governing bodies over the last decade. Thus, there is an onus on researchers and medical departments to develop a better understanding of the physical characteristics and demands, and the health and performance needs of female soccer players. In this review, we discuss the current research, as well as the knowledge gaps, of six major topics: physical demands, talent identification, body composition, injury risk and prevention, health and nutrition. Data on female talent identification are scarce, and future studies need to elucidate the influence of relative age and maturation selection across age groups. Regarding the physical demands, more research is needed on the pattern of high-intensity sprinting during matches and the contribution of soccer-specific movements. Injuries are not uncommon in female soccer players, but targeting intrinsically modifiable factors with injury prevention programmes can reduce injury rates. The anthropometric and physical characteristics of female players are heterogeneous and setting specific targets should be discouraged in youth and sub-elite players. Menstrual cycle phase may influence performance and injury risk; however, there are few studies in soccer players. Nutrition plays a critical role in health and performance and ensuring adequate energy intake remains a priority. Despite recent progress, there is considerably less research in female than male soccer players. Many gaps in our understanding of how best to develop and manage the health and performance of female soccer players remain.

## Key Points

**Table:** 

The use of micro-electrical mechanical system (MEMS) devices in the sport has enabled soccer-specific movements to be more accurately described, and the use of MEMS in female soccer research is now warranted.
Identifying characteristics that are responsible for both de-selection from the elite academy pathway and re-selection at lower levels of soccer is of relevance for elite female youth soccer players.
Organisations should develop a professional framework for support staff to encapsulate clear guidelines and processes on body composition assessment, interpretation and activation for female soccer players.
Previous injury is a key intrinsic risk factor for future injury in female soccer players; modifiable risk factors are of interest, as action can be taken to reduce their impact on the number of initial injuries.
Female soccer teams might benefit from monitoring energy availability (EA) at times when sub-optimal EA is more likely, and practitioners should educate players on the negative consequences of low EA.
Intervention-based studies utilising different nutritional strategies are lacking in female soccer players. More research is warranted, and can be facilitated within professional clubs providing the barriers to such research are recognised and overcome.

## Introduction

Interest in female soccer has grown exponentially in recent years [1]. Financial support from the Union of European Football Associations (UEFA) has trebled [2], and participation rates over the last 10 years have grown by a third [3]. Worldwide, the Fédération Internationale de Football Association (FIFA) is committed to increase the number of female soccer players from ~ 13.3 million (2019) to 60 million by 2026 [1].

In professional female soccer, as well as the male equivalent, it is incumbent that coaches and support staff protect the health and well-being of players, as well as optimise their performance. But in contrast to professional men’s soccer, and largely due to the explosion in female participation, science has struggled to keep pace with the demand for evidence-based studies to inform practice [4], as female players transition from amateur to professional level. In a recent scoping review, it was found that the most popular publication themes related to women’s football are sports medicine, strength and conditioning and sociology [4]. Therefore, the aim of this review is to discuss research addressing other major topics relating to female soccer players: physical demands of match-play, talent identification, anthropometric and physical characteristics, injury risk and prevention, health and nutrition. By compiling such a review, and dissecting these important topics, the authors hope to raise awareness of outstanding research questions and accelerate answers, in an effort to better understand and optimise the health and performance of female soccer players. Throughout this review, the sport will be referred to as “female soccer” and the terminology “player” refers to female soccer players, unless otherwise stated. This review will focus on research conducted in female soccer players, with a focus on elite level players, where possible. If data are not available on soccer players, extrapolations will be made from other existing studies conducted in female athletes.

## Physical Demands of Female Soccer

At any level of soccer, effective tactical plans require co-ordinated movement of players around the pitch. The performance of technical skills to complete key match actions complements these movements. These factors determine the physical requirements of soccer. The available research on the demands of female soccer has primarily focused on describing the volume, intensity and activity patterns of players [5]. This approach stems from the desire to determine the metabolic demands of game play. The total distance covered during a game is indicative of the volume of activity completed by players and despite some variation, elite players generally cover ~ 10 km during a match [6–10]. While these values may be useful in providing a global estimation of the overall movement demands, the intensity at which this activity is completed is of greater importance. More specifically, it is the amount of the activity that is completed at high and/or maximal running speeds that is key [5]; as such, these activities may be better related to team success [11].

The distance covered in high-intensity actions is typically determined by applying specific speed thresholds to players' movements, and then calculating the amount of activity that exceeds the relevant limit [12]. Both the methodology to determine speed thresholds (individual vs generic, e.g. Scott and Lovell [13] and Datson et al. [14]; and performance vs statistical, Bradley and Vescovi [15] and Park et al. [16]), and a resulting lack of a definitive approach to identify high-intensity actions, result in ambiguity in this area and make it difficult to generate a detailed consensus of the data [15]. These challenges are further compounded by variations in the accuracy of measurement associated with the different methodologies/technologies used to collect data [12]. However, recent publications by Scott and Lovell [13] and Park et al., [16] would seem to imply that methodological approaches may not influence data to the extent suggested in previous literature. The available data (see Table 1) are, however, variable for high-intensity activity and sprinting. While the sources of this variation have not specifically been identified it is likely that a combination of the specific population studied, the approach to data collection and analysis and the context of the games (i.e. home and away) used in the sample will be contributing to the factors that could play a role. Despite this variation in the contribution to the overall activity, high-intensity running and sprinting are crucial components (22–28% of the total match distance covered [17]) of the physiological demands of the sport (due to the nature of their involvement in specific crucial match actions and off the ball running), and will necessitate the involvement of additional metabolic and physiological resources (e.g. the anaerobic energy system). This will, in turn, have implications for the types of preparation exercises that are included in training programmes.

**Table 1:** Methodological details and outcome measures related to high-intensity actions from studies post-2014 (for research before 2014, see [5]). Terminology has been altered in some cases from that used in the original reference to provide consistent terminology for the table

Reference	*N*	Playing level	Measurement technology	Speed thresholds	High-intensity running distance (m)	Sprinting distance (m)
Scott et al. [173]	220	National Women’s Soccer League USA	Global positioning system	High-speed running > 19.0 km.h^−1^ Sprint > 22.5 km.h^−1^	350–666	98–248
Mara et al. [174]	12	Australian National League	Optical tracking	High-speed running 12.2—19.1 km.h^−1^ Sprint > 19.4 km.h^−1^	1772–2917	417–850
Ramos et al. [8]	45	Brazilian National U17, U20 and Senior	Global positioning system	High-speed running 15.6—20 km.h^−1^ Sprint > 20 km.h^−1^	347–840	138–379
Datson et al. [14]	107	Senior International players	Optical tracking	High-speed running > 19.8 km.h^−1^ Sprint > 25.1 km.h^−1^	534–920	111–221
Trewin et al. [21]	45	Senior International players	Global positioning system	High-speed running > 16.5 km.h^−1^ Sprint > 20 km.h^−1^	661–1191	No data provided
Sausaman et al. [9]	23	College players	Global positioning system	High-speed running > 15 km.h^−1^ Sprint > 18 km.h^−1^	840–1333	267–633
Ramos et al. [175]	12	U20 International players	Global positioning system	High-speed running 15.6–20 km.h^−1^ Sprint > 20 km.h^−1^	508–854	113–331
Jagim et al. [6]	25	College players	Global positioning system	High-speed running 15.0–18.99 km.h^−1^ Sprint > 19 km.h^−1^	658–916	140–403

The pattern in which high-intensity activity is completed is a fundamental factor in the overall physiological cost of the exercise. This has led to an interest in the evaluation of repeated sprint activities. While there are some discrepancies in the specific definition of repeated sprint activity, the majority of papers use broad classifications that include multiple high-speed running and/or sprinting bouts within a given recovery period (e.g. Datson et al. [18]). The literature indicates that the frequency of such bouts in games is between 1 and 25 bouts [18–20] and this range may suggest that the ability to complete this type of high-intensity activity with short recovery is not a crucial component of the demands of the game. These data, which largely reflect the average requirements may not, however, demonstrate the “worst-case scenarios” (i.e. the maximum amount of repeated sprint activity that could be completed by a player within a game) that players face [18]. Additional research is needed before the relative importance of activity patterns, and their implications for training and preparation, are fully understood within the female game. This research could involve identifying specific situations within games that are associated with the highest demands, as well as determining actions that surround critical events during a match.

Match activity for high-speed running [coefficient of variation (CV) = 33%] and sprint-efforts (CV = 53%) is inherently variable between games in female soccer [21]. This variation is largely a result of factors that seem to impact the generic activity profiles of players, such as, environmental conditions [22, 23], playing position [14, 17], level of play [8, 24, 25] and other contextual factors including opponent quality [7], surface [26] and team tactics [11]. These variations in activity likely reflect the impact of external factors that influence the tactical and technical structure and tempo of the game, though additional research is needed to establish the extent of this variability in more recent samples of match-play. Irrespective of the variation in overall activity demands, match-play per se disrupts normal levels of physiological function as performance (e.g. sprinting, sport-specific movements and strength), biochemical markers in the blood (e.g. creatine kinase) and muscle soreness are all altered following competition [27–29]. This would seem to suggest that even for well-trained players, the demands of the game exceed those associated with training and that this stress results in a temporal disruption that limits relevant aspects of physiological function [30]. Such observations may be purely a consequence of the challenge faced by individual players to adapt to an ever-changing game stimulus or may alternatively suggest that current approaches to training may not be optimised for repeated game performance.

A focus on the description of the demands, from a locomotive perspective, neglects other activities that may be important in influencing the overall physical demands. The activity profile in female soccer is characterised by a large number of activity changes in the locomotor pattern (> 1300) [24]. These activity changes are a consequence of the repeated accelerations, decelerations and changes of direction that are required by players to respond to the changing nature of the game and to perform technical/and tactical actions effectively. Accelerations, decelerations and changes of direction, until relatively recently, have not been easily quantifiable. The introduction, however, of micro-electrical mechanical system (MEMS) devices into the sport has enabled such movements to be more accurately described [8]. Observations of lower levels of variability in such outcome measures [21, 31], as well as the potential for these actions to provide an indication of the “mechanical load” [12, 32] associated with soccer-specific movements, make such variables important in future descriptions of the activities in the game. Technology included in MEMS devices may also be important in describing the demands associated with other game-specific actions such as the technical skills of kicking, jumping and tackling. These will be associated with a physiological cost though few studies [33] are currently available in female soccer that describe either the extent or the demand associated with such actions. An understanding of both the consequences of changes in locomotor pattern and the impact of specific technical actions on the physical demands of the women’s game would represent a valuable research focus in future years.

## Talent Identification

As participation rates increase, the talent pool from which to draw players has expanded. This expansion, coupled with increased competition, prompts a need for elite level soccer clubs to develop methods of talent identification and development. These methods should reflect the specific demands of the female game and maximise the recruitment and development of the most talented players [34, 35]. However, the proportion of players likely to progress through the development pathway is small [35]. Therefore, identifying the characteristics that are responsible for both de-selection from the elite academy pathway and re-selection at lower levels of soccer has particular relevance for most elite youth soccer players.

A paucity of literature exists in the area of talent identification of the female elite youth soccer player [34]. Despite the majority of existing data being cross-sectional and mono-disciplinary in nature (Table 2), several common themes have emerged. High-intensity endurance capacity appears to have some prognostic power in identifying young players that have reached the elite level (national team/first division) of female soccer [36, 37] or the potential to reach that level [38]. In addition, the slalom dribbling test [39] has the capability to differentiate between players that reached youth national team vs regional academy level (Table 2). However, sprint duration does not seem to discriminate for talent identification purposes [36, 37, 39, 40] (Table 2).

**Table 2:** Physiological and motor determinants of future playing success in elite female youth soccer players

Reference	*N*	Age (years)	Playing level	Determinants of performance	Major findings
Hoare and Warr [38]	59	15–19	Individual sport and non-soccer team sport players recruited into a soccer training camp	VJ, 20 m PST, 20 m linear sprint. 505 Agility Test	17 selected players from the 59 demonstrated VJ height in the 80th percentile and maximal aerobic power in the 90th percentile compared to the Australian population values for 15 year olds. 20 m sprint time faster (3.47 s) than the population average (3.64 s) at 15 years of age
Datson et al. [36]	228	12.7—15.3	Elite Performance Camps (English FA)	CMJ, 30 m linear sprint, YYIR1	Higher YYIR1 (2040 m) score more likely (47–82%) to be selected into U17–U20 International Squad
Höner et al. [39]	499	11.4	German Soccer Talent Programme	Sprint time, agility, dribbling, ball control and shooting	Dribbling was the most relevant motor predictor for German Youth National Team Selection
Vescovi et al. [40]	414	12–21	High club-level juniors and NCAA, Div 1 US College Players	CMJ, Illinois Agility Test (modified) and 36 m RST	No evidence of mean linear sprint speed (9.1 m) differences across all age groups
Mujika et al. [37]	34	17–24	Elite senior female players from the Spanish Super Liga and junior players from the Spanish 2nd Division	CMJ, YYIR1, linear sprint 15 m, ball dribbling 15 m	Elite players (1224 m) superior to Junior (826 m) in YYIR1. No difference in 15 m sprint time between senior and junior players

*VJ* vertical jump, *PST* progressive shuttle test, *CMJ* counter movement jump, *YYIR1-Yo-Yo* intermittent recovery level 1 test, *RST* repeated sprint test

These studies are valuable, as they provide some initial insight into primarily physical or motor determinants required for success as an elite, female youth soccer player using a single time-point of analyses (Table 2). The major limitation of this methodological approach is that cross-sectional data are limited in their capacity to provide a prediction of future success as adult soccer players. Talent development is a non-linear, dynamic construct, so serial measurements of performance are needed over time to truly understand the trajectory of the elite youth soccer player’s development. Incorporating an array of potential soccer performance determinants into a longitudinal evaluation of the player appears to be the optimal approach to understand talent identification and development in an elite female youth soccer environment.

Two studies to date have adopted this strategy [35, 38]. Using a quasi-experimental approach, researchers recruited non-soccer playing individuals aged between 15 and 19 years based on a composite of physical performance and skill based metrics to form a soccer team [38]. These researchers subsequently tracked the performance of the team (wins and losses) and individual player success (final playing destination) over a period of 2 years. There was evidence of sustained team success (significant winning record) across the first season. Two years later, six players from the original cohort had been selected to play in Australian Premier League clubs. Unfortunately, there was no tracking of any physical performance or skill metrics that mediated the progression to elite level for these players.

Subsequently, a longitudinal study was conducted by the German Football Federation [35] in an attempt to provide insight into this question. Skill (dribbling, passing and target shooting) and physical fitness (20 m linear sprints and a slalom agility run without the ball) variables were evaluated over a 4-year period in adolescent players. The final football destination, either professional (6.2%) or non-professional (93.8%) club, of these players was evaluated 4 years after completion of testing. Players who were ultimately affiliated with professional clubs were ~ 1 s faster on the sprint, passing and agility drills than their peers who played at non-professional levels. These differences were apparent from U12 and through to U15. The rate of improvement for these measures in both groups over time was non-linear and the authors concluded that motor performance had some prognostic relevance over the final football destination of these adolescent players. On their own, however, these metrics did not have sufficient predictive power to determine success in adult soccer.

It is noteworthy that all the metrics used in the aforementioned study were closed skill tests (sprinting, dribbling and shooting) and concern exists with regard to how soccer specific these tests are. Furthermore, none of the contextual factors (opposition players and teammates) were present that can impact performance [35] and no maturity status measurements were obtained. Consequently, it was not possible to differentiate between the influence of growth and maturation from training on the changes in these motor performance metrics.

The combination of cross-sectional and longitudinal studies evaluated in this review has provided a foundation for talent identification and development in elite youth players, but more work is required. The following topics represent possible areas for future work in elite youth soccer. For example, evaluating the influence of the relative age effect and the maturation-selection phenomenon across all age groups in elite youth soccer. In addition, exploring the prognostic power of agility, co-ordination and perception and cognition and further refining the predictive model created by Leyhr et al. [35] are key areas of future development. Finally, exploring other contextually appropriate methods to evaluate talent identification and development such as modified small-sided games would be useful [41, 42] and the application of constructs that investigate key psychological traits for future success such as resilience and perseverance is warranted.

## Anthropometric and Physical Characteristics

The anthropometric and physical characteristics of athletes are factors that can contribute to successful performance and health status [43]. Acquiring information on professional female soccer players could be valuable for coaches and practitioners, and may help to inform training and talent development processes. The anthropometric and physical characteristics of players competing in the national team or highest national league are presented in Table 3. Players ranged between 19 and 26 years of age, 1.61–1.70 m in stature, 56.6–65.1 kg in body mass and had a body fat percentage of 14.5–22.0%. Related body composition ranged from 11.5 to 15.3 kg for fat mass, 42.5–49.5 kg for lean mass and a total bone mineral density of 1.26–1.30 g/cm^2^ (Table 4). When comparing the data from the last 5 years with the previous 15 years (stature: 1.67 cf 1.67 m; body mass: 61.3 cf 62.6 kg; combined data in Table 3), players’ stature and body mass have remained similar over time [5, 44]. Values for age, stature and body mass are in-line with the information of 552 players from 24 countries participating in the 2019 FIFA World Cup [11]. The large range (age: 16–41 years, stature: 1.48–1.87 m, body mass: 46–88 kg [11]) reiterates the heterogeneity among top-level soccer players [5]. Therefore, coaches, players and practitioners should acknowledge that adopting specific anthropometric and body composition targets is currently unjustified.

**Table 3:** Anthropometric data from elite adult soccer players competing in the national team or highest national league. Data collated from 2000 to 2020

Reference	*N*	Country	Standard and time-point	Age (years)	Stature (m)	Weight (kg)	% body fat	Fat mass (kg)	Lean mass (kg)
Andersson et al. [25]	17	Sweden, Denmark	National team In-season	27 ±1	1.68 ± 0.02	61.0 ± 1.4	–	–	–
Andersson et al. [176]	21	Sweden	Highest division In-season	24.3 ± 4.9	1.70 ± 0.02	62.9 ± 4.9	–	–	–
Andersson et al. [28]	17	Sweden, Norway	Highest division	22.6 ± 4.2 21.6 ± 2.6	1.67 ± 0.06 1.67 ± 0.05	63.3 ± 7.1 65.0 ± 4.6	–	–	–
Bellver et al. [177]	92 (46 for DXA)	Spain	1st and 2nd teams Futbol Club Barcelona Season period unknown	22.0 ± 5.2	1.66 ± 0.06	59.9 ± 6.4	–	14.6 ± 3.9	42.5 ± 4.5 A: 4.2 ± 0.6 L: 15.5 ± 1.6
Brewer et al. [178]	27	USA	Highest division (NCAA D1) Pre-season	20.0 ± 1.4	1.68 ± 0.06	65.1 ± 7.1	–	–	–
Can et al. [179]	14	Turkey	Highest division Pre-season	20.7 ± 2.1	1.62 ± 0.06	56.6 ± 5.0	19.8 ± 0.7	–	–
Castagna and Castellini [180]	21	Italy	National team In-season	25.8 ± 3.9	1.67 ± 0.04	59.9 ± 3.8	–	–	–
Clark et al. [181]	14	USA	Highest division (NCAA D1) Pre-season Post-season	19.7 ± 0.7 20.0 ± 0.9	1.66 ± 0.05 1.66 ± 0.05	62.0 ± 4.8 61.6 ± 4.7	16.4 ± 2.4 16.1 ± 2.8^a^	–	–
Emmonds et al. [182]	10	England	Highest division (WSL1) Start of season	25.4 ± 7.0	1.67 ± 0.05	62.6 ± 5.1	21.3 ± 3.87^b^	12.9 ± 2.3	46.3 ± 4.5
Fields et al. [45]	110 19 46 32 12	USA	Highest division (NCAA D1) Forward Midfield Defender GK Off-season	18–24	–	63.2 ± 7.9 62.2 ± 8.4 61.1 ± 6.8 63.3 ± 6.8 72.1 ± 8.3	22.6 ± 5.5^c^ 22.2 ± 5.8 21.1 ± 5.5 23.6 ± 5.0 26.6 ± 4.7	14.5 ± 4.5^c^ 13.9 ± 4.4 13.0 ± 3.9 15.0 ± 4.2 19.4 ± 5.3	48.7 ± 5.4^c^ 48.2 ± 6.2 48.2 ± 6.2 48.1 ± 5.2 52.7 ± 4.2
Gravina et al. [30]	14	Spain	Highest division Season period unknown	25 ± 5	–	61 ± 7.4	15.5 ± 2.9^d^	–	–
Ingebrigsten et al. [48]	29 8 8 10 3	Norway	Highest divisions Forward Midfielder Defender GK Pre-season	20.8 ± 3.7	1.66 ± 0.05 1.64 ± 0.04 1.65 ± 0.04 1.69 ± 0.05 1.69 ± 0.08	60.7 ± 6.6 58.4 ± 5.2 61.3 ± 7.3 62.5 ± 7.3 59.5 ± 7.2	–	–	–
Krustrup et al. [27]	23	Denmark	Highest division In-season	23 (18–29)	1.69 (1.59–1.80)	60.1 (53.3–69.5)	18.5 (12.7–27.6)	–	–
Krustrup et al. [183]	14	Denmark	Highest division In-season	24 (19–31)	1.67 (1.56–1.80)	58.5 (49.0–70.7)	14.6 (9.3–21.9)	–	–
Manson et al. [3]	51	New Zealand	National team In-season	15.6 ± 1.0	1.64 ± 0.05	58.0 ± 5.48	–	–	–
Mara et al. [184]	17	Australia	Elite National League team Pre-season Post-season	–	1.73 ± 0.06	64.3 ± 5.9 65.2 ± 6.8	21.5 ± 6.0 22.4 ± 6.4	–	73.8 ± 6.2 (%) 72.8 ± 6.5 (%)
Milanovic et al. [185]	22	Serbia	National team	24.0 ± 4.5	1.69 ± 0.07	61.4 ± 6.0	–	–	–
Minett et al. [186]	24	USA	Highest division (NCAA D1) Pre-season Post-season	19 ± 0.2	1.65 ± 0.10	64 ± 1.5	22 ± 0.7^b^	14 ± 0.8^b^	48 ± 0.9^b^
Moss et al. [129]	13	England	Highest division (WSL1) In-season	23.7 ± 3.4	1.69 ± 0.08	63.7 ± 7.0	17.8 ± 4.4^b^	11.5 ± 3.5^b^	49.5 ± 5.3 LL: 8.5 ± 1.1 RL: 8.8 ± 1.0^b^
Mujika et al. [37]	17	Spain	Highest division Pre-season	23.1 ± 2.9	1.65 ± 0.04	56.8 ± 5.7	–	–	–
Parpa and Michaelides [187]	18	Cyprus	Highest division End of season	23.6 ± 4.3	1.65 ± 0.05	58.3 ± 6.5	19.8 ± 3.5^e^	–	–
Risso et al. [188]	22	USA	Highest division (NCAA D1)	S: 20.4 ± 1.3 NS: 20.1 ± 1.2	1.67 ± 0.05 1.66 ± 0.06	59.8 ± 7.1 62.8 ± 6.6	–	–	–
Sedano et al. [47]	100	Spain	Highest division In-season	22.1 ± 1.1	1.61 ± 0.06	57.7 ± 7.5	20.1 ± 5.5^f^	–	–
Sjokvist et al. [189]	14	USA	Highest division (NCAA D1) Season period unknown	20.3 ± 2.3	1.68 ± 0.05	61.9 ± 6.5	20.9 ± 3.4^f^	–	–
Stanforth et al. [190]	47	USA	Highest division (NCAA D1) 3-year average Pre-season Post-season	-	1.66 ± 0.01	62.5 ± 0.5 62.3 ± 0.7 62.7 ± 0.7	24.1 ± 0.4^b^ 24.0 ± 0.5 24.2 ± 0.5	15.2 ± 0.3^a^ 15.1 ± 0.4 15.3 ± 0.4	44.4 ± 0.3^b^ 44.4 ± 0.5 44.5 ± 0.5
Vescovi et al. [46]	64 17 18 21 8	USA	Highest division (NCAA D1) Forward Midfielder Defender GK	19.8 ± 1.2 19.5 ± 1.1 20.0 ± 1.3 19.9 ± 1.1 19.6 ± 1.1	1.68 ± 0.06 1.68 ± 0.07 1.66 ± 0.06 1.70 ± 0.04 1.70 ± 0.06	64.8 ± 5.9 64.5 ± 5.8 61.3 ± 4.7 67.0 ± 6.7 66.4 ± 1.9	–	–	–
Vescovi and McGuigan [191]	51	USA	Highest division (NCAA D1)	19.9 ± 0.9	1.68 ± 0.06	64.8 ± 5.9	–	–	–

*A* arms, *L* legs, *H* hip, *FN* femoral neck, *T* trochanter, *D* diaphysis, *NCAA* National Collegiate Athletic Association, *S* starters, *NS* non-starters. Where standard deviation is not available, the range has been included

^a^Analysed by hydrostatic weighing

^b^Analysed by DXA

^c^Analysed by air-displacement plethysmography

^d^Method unknown

^e^Analysed by bio-electrical impedance

^f^Analysed by skinfolds

**Table 4:** The bone mineral content (g) and density (g/cm^2^) of elite adult soccer players

Reference	*N*	Country	Standard and time-point	Age (years)	Bone mineral content (g)^a^	Bone mineral density (g/cm^2^)^a^
Bellver et al. [177]	46	Spain	First and second teams of Futbol Club Barcelona Season period unknown	22.0 ± 5.2	2.7 ± 0.3	1.26 ± 0.1 H: 1.251 ± 0.14 T: 1.039 ± 0.14 D: 1.453 ± 0.18 L1-L4: 1.34 ± 0.16 FN: 1.24 ± 0.14
Minett et al. [186]	24	USA	Highest division (NCAA D1) Pre-season Post-season	19.0 ± 0.2	H: 37.0 ± 1.0 FN: 5.0 ± 0.1 H: 37.3 ± 1.1 FN: 5.0 ± 0.1	H: 1.13 ± 0.0 FN: 1.02 ± 0.0 H: 1.13 ± 0.0 FN: 1.03 ± 0.0
Moss et al. [129]	13	England	Highest division (WSL1) In-season	23.7 ± 3.4	–	1.3 ± 0.1 H: 1.4 ± 0.1

*H* hip, *FN *femoral neck, *T *trochanter, *D *diaphysis, *L1–L4 *lumbar 1–4, *NCAA* *D1 *National Collegiate Athletic Association Division 1, *DXA* dual-energy X-ray absorptiometry, *WSL1 *Women’s Super League 1st Division

^a^Analysed by DXA

Positional differences of elite players have been assessed in a small number of studies [45–48]. Similarities between positions for stature and body mass have been reported [46, 48]. Nevertheless, the ~ 4–5 cm mean difference in stature between goalkeepers (tallest) and forwards (shortest) and the ~ 4–6 kg mean difference in body mass between defenders (heaviest) and midfielders (lightest) is noteworthy [46, 48]. Most recently, body fat percentage, body mass and fat mass were found to be higher in goalkeepers compared to other positions in Division 1 National Collegiate Athletic Association (NCAA) players, with no differences between outfield positions [45]. In contrast, a study in Spanish players [47] reported anthropometric differences between all playing positions. However, the absolute differences for elite players were unavailable as values were combined with non-elite players. Future research should determine if specific anthropometric profiles evolve to become characteristic of specific playing positions.

In female soccer, as in other sports, organisations may use body composition assessment to aid interpretation of health and performance-relevant results and inform subsequent exercise and dietary interventions [49]. Ensuring that assessment is useful and safe for players requires practitioners to be aware of possible problems that could occur when the focus on body mass and body composition is over-emphasised [49]. In professional female soccer, player interviews revealed personal accounts of being “over tested” which was suggested to alter eating habits, specifically via the team avoiding carbohydrates after intense training sessions [50]. Although it is not clear whether such instances are widespread in female soccer, cultural problems around body composition and body image have been identified in women’s sport [51]. Therefore, there is a need to create and maintain healthy practices around body composition that commit to maintaining or improving the long-term health of players [51].

Although not soccer specific, best practice protocols and guidelines on safe standards for assessment and dissemination of results are available [49, 51]. Ensuring standardisation of all protocols including method, tester, frequency, hydration testing, the process for requesting body composition assessment and data dissemination is essential [49]. While decisions on the specifics of such protocols are highly dependent on the context within which the organisation operates, it is suggested that where appropriate the method of assessment should be dual-energy X-ray absorptiometry (DXA) [52] or skinfolds [International Society for the Advancement of Kinanthropometry (ISAK)]. These have been identified as appropriate methods for detecting body composition changes due to an intervention [53]. As well as providing repeatable estimates of fat and lean mass, DXA has the added benefit of estimating bone mineral mass [54]. Guidance on frequency of assessment states that there should be at least 2–6 months between assessments, although if DXA is completed, two times per year is appropriate [49]. Promoting a positive culture around body composition (i.e. via eliminating environments that focus on body shaming) and providing education about the negative effects of chronic low energy availability (see Sect.6.5 section for more information) are also crucial to maintaining healthy practices and require commitment from all staff and players [51]. Appropriate protocols for screening (e.g. Eating Disorder Inventory, Eating Attitudes Test-26 and clinical interviews) and subsequent management for underweight and/or individuals with unhealthy attitudes/behaviours should be developed by personnel with appropriate expertise (e.g. sports nutritionists, dieticians, medics, psychologists) [49, 53]. Therefore, it is recommended that organisations develop a professional body composition framework (see Meyer et al. [49]). The framework should be understood by all staff and establish clear guidelines on body composition assessment as well as appropriate framing of the results to best support the health, well-being and performance goals of the player.

## Injury Incidence, Risk and Prevention

To embed successful injury management strategies in female soccer, the first steps are to understand the epidemiology of injury incidence and burden. Two recent systematic reviews and meta-analyses in female adult and youth soccer [55, 56] have reported an overall injury incidence of 6.1 and 7.1 injuries/1000 h playing time, respectively. In adult and youth players, there is a six- to sevenfold higher incidence rate in matches (adults: 19.2, youth: 14.9) compared with training (adults: 3.5, youth: 2.9). These rate discrepancies potentially indicate that training may be purposefully controlled to avoid unnecessary injuries or, as recent data suggest, training does not replicate match-play to provide robustness and readiness to perform in competitive play [57]. Limited data suggest that injury incidence is greater during tournament play than the regular season (24.6 vs 17.3) and this has been attributed to greater match congestion, reduced recovery times and accumulated fatigue [56]. Injuries were more likely to be traumatic and non-contact in nature (4.6 injuries/1000 h exposure) compared with overuse injuries (1.6 injuries/1000 h exposure). The lower extremities were the most likely sites of injury (4.8 injuries/1000 h exposure) with the ankle (1.1 injuries/1000 h exposure) and knee (1.1 injuries/1000 h exposure) having the greatest incidence. The most likely types of injuries in adult players were muscle and tendon injuries (1.8 injuries/1000 h exposure) followed by joint (non-bone) and ligament injuries (1.1 injuries/1000 h exposure) [56]. For youth players, the greatest types of injuries were joint (non-bone) and ligament (2.4 injuries/1000 h exposure) [55]. In terms of injury burden, most time loss injuries are classified as slight or minimal [1–3-day loss] (mean = 2.2 days). However, female soccer players sustain a greater number of severe injuries [time loss > 28 days] compared with male players [56]. This might be attributed to the greater incidence of ligament injuries sustained by female players, especially to the anterior cruciate ligament (ACL). Analysis indicates a significantly higher injury incidence rate in players participating in lower leagues (outside of FIFA's top 15 leagues) suggesting that those with more resource and greater levels of professional status have lower incidence rates. Tables 5 and 6 provide details of the injury incidence as recently cited in Robles-Palazón et al. [55] and López-Valenciano et al. [56].

**Table 5:** Incidence of injury in youth female soccer players. Adapted from López-Valenciano et al. [56] and Robles-Palazón et al. [55], with permission

Reference	Study duration (weeks)	Age (range, years)	Teams (players)	Exposure (h)			Injuries			Incidence (per 1000 h)		
Continent (or event)/year/level of play				Overall	Training	Match	Overall	Training	Match	Overall	Training	Match
Andreasen et al. [192] IT/1991/EL	1	U19 (10–19)	– (3321)	–	–	8890	–	–	39	–	–	4.4
Backous et al. [193] NA/–/MI	3	U17 (6–17)	– (458)	10,094.3	–	–	107	–	–	10.6	–	–
Clausen et al. [194]^a^ EU/2012/MI(T), EL(a), SEL(b)(c)	20(a)	U18 (15–18)	– (–)	6434.0	–	–	59	–	–	9.2	–	–
	20(b)	U18 (15–18)	– (–)	6811.0	–	–	63	–	–	9.2	–	–
	20(c)	U18 (15–18)	– (–)	13,761.0	–	–	140	–	–	10.2	–	–
	20(T)	U18 (15–18)	32 (438)	27,746.0	–	–	269	–	–	9.7	–	–
Hägglund et al. [195] (d)^a^ EC/2006/EL	2	U19 (U19)	8 (144)	1707.0	1210.0	497.0	19	6	13	11.1	5.0	26.2
Hägglund et al. [195] (e)^a^ EC/2007/EL	2	U19 (U19)	8 (144)	1407.0	906.0	501.0	12	1	11	8.5	1.1	22.0
Hägglund et al. [195] (f)^a^ EC/2008/EL	2	U19 (U19)	8 (145)	1635.0	1121.0	514.0	8	2	6	4.9	1.8	11.7
Junge et al. [196]^a^ WC/2008–12/EL	9	U17 (U17)	48 (1008)	–	–	3168.0	–	–	68	–	–	21.5
Le Gall et al. [77]^a^ EU/1998-06/EL	312	U19 (15–19)	– (119)	97,325.0	87,530.0	9795.0	619	400	219	6.4	4.6	22.4
Lislevand et al. [197]^a^ AF/2008/SEL	0.3	U13 (≤ 13)	37 (433)	–	–	431.0	–	–	5	–	–	11.6
		U16 (13–16)	14 (213)	–	–	403.0	–	–	1	–	–	11.7
		U16 (≤ 16)	51 (646)	–	–	834.0	–	–	6	–	–	7.2
Schmidt-Olsen et al. [198] IT/1984/EL	1	U13 (9–13)	– (361)	–	–	13,043.5	–	–	7	–	–	0.5
		U16 (14–16)	– (732)	–	–	1943.0	–	–	49	–	–	25.2
		U19 (17–19)	– (232)	–	–	635.6	–	–	13	–	–	20.9
Söderman et al. [65] EU/1996/SEL	28	U19 (14–19)	10 (153)	11,689.2	–	–	79	–	–	6.8	–	–
Soligard et al. [199]^a^ Control EU/2007/–	32	U17 (13–17)	– (837)	45,428.0	31,086.0	14,342.0	215	74	138	4.7	2.4	9.6
Steffen et al. [62]^a^ Control EU/2005/–	32	U17 (13–17)	51 (947)	65,725.0	–	-	241	–	–	3.7	–	–

^a^In reference column: study was implemented according to the 2006 consensus statement for epidemiological studies in football. Letters in parentheses indicates different cohorts in the same study; (T) indicate the total sample of the study

*EL* elite, *SEL* sub-elite, *MI* mixed (elite and sub-elite), *F* female, *U* under, *EU* Europe, *NA* North America, *SA* South America, *AS* Asia, *AF* Africa, *OC* Oceania, *EC* European Championship, *ET* European Tournament, *IT* International Tournament; WC: World Championship

**Table 6:** Incidence of injury in adult female soccer players. Adapted from López-Valenciano et al. [56] and Robles-Palazón et al. [55], with permission

Reference	Study duration (weeks)	*N* Teams	Exposure (h)			Injuries			Incidence (per 1000 h)		
Country/tournament		(Players)	Overall	Training	Match	Overall	Training	Match	Overall	Training	Match
Babwah [200] Trinidad and Tobago—2009	16	16 (320)	–	–	941	–	–	29	–	–	27.6
Becker et al. [201] Germany—2000/2001	36	12 (254)	86,746	–	–	216	–	–	2.5	–	–
Ekstrand et al. [202] Sweden—2003/2008	256	5 (154)	48,404	–	–	314	–	–	6.5	–	–
Elias et al. [203] USA—2011	390	– (–)	–	–	21,805	–	–	232	–	–	10.6
Engström et al. [204] Sweden	39	2 (41)	6500	4142	2041	78	29	49	12.0	7.0	24.0
Faude et al. [205] Germany—2003/2004	38	9 (165)	35,310	30,195	5115	241	–	–	6.9	–	–
Fuller et al. [206] USA—2005/2006	96	136 (–)	324,751	280,496	44,255	1720	774	946	5.3	2.8	21.4
Gaulrapp et al. [207] Germany	44	12 (254)	75,438	67,056	8382	246	91	155	3.3	1.4	18.5
Giza et al. [78] USA—2001/2002	78	8 (202)	89,637	–	–	173	–	–	1.9	1.2	12.6
Hägglund et al. [195] Switzerland/U19 EC—2006	2	8 (144)	1707	1210	497	23	9	14	13.5	7.4	28.2
Hägglund et al. [195] Iceland/U19 EC—2007	2	8 (144)	1407	906	501	12	1	11	8.5	1.1	22.0
Hägglund et al. [195] France /U19 EC– 2007	2	8 (145)	1635	1121	514	8	2	6	4.9	1.8	11.7
Jacobson et al. [67] Sweden—2005	34	18 (253)	23,854	11,428	10,000	229	96	133	9.6	8.4	13.3
Jacobson et al. [67] Sweden—2000	43	12 (195)	51,522	44,815	8345	237	121	116	4.6	2.7	13.9
Junge et al. [196] FIFA WCs—1999/2011	16	64 (1312)	–	–	4224	–	–	95	–	–	22.5
Junge et al. [196] OG Tournaments—2000/2012	12	128 (828)	–	–	2904	–	–	81	–	–	27.9
Junge et al. [196] FIFA U19/U20 WCs—2002/2012	12	360 (1812)	–	–	5940	–	–	175	–	–	29.5
Larruskain et al. [208] Spain—2010/2015	260	1 (35)	25,394	21,850	3544	160	75	80	6.3	3.4	22.6
Maehlum et al. [209] IT Norway—1984	1	332 (–)	–	3440	8218	–	–	145	–	–	17.6
Nilstad et al. [210] Norway—2009	32	9 (159)	66,387	53,157	12,694	232	135	97	3.5	2.5	7.6
Östenberg et al. [75] Sweden—1996	281	8 (123)	9745	7027	2727	65	26	39	6.7	3.7	14.3
Owoeye et al. [211] Nigeria—2012	4	10 (300)	–	–	759	–	–	6	–	–	7.9
Tegnander et al. [212] Norway—2001	28	10 (181)	30,619	–	3663	189	100	89	6.2	3.7	24.3
Waldén et al. [213] England/ EC—2005	2	8 (160)	1820	–	507	18	3	15	9.9	2.3	29.6

Given the complexities in growth and maturation and an increase in injury incidence linked to peak height velocity [58], it is surprising that there are limited data in youth players. As injury burden is increased for ligament injuries, it is important to understand the risk factors and subsequent injury management strategies that should be embedded in the female soccer player pathway.

### Risk Factors

The cause of injury in soccer is often complex and affected by a multifaceted interaction of risk factors [59]. Risk factors may be classified as intrinsic (i.e. athlete-related) or extrinsic (i.e. environment-related) or indeed modifiable or non-modifiable [60]. It is vital to identify risk factors and injury mechanisms to establish potential preventative strategies [61]. Previous injury is widely documented as a key intrinsic risk factor for future injury in both youth [62–64] and senior [65–69] players. An increased injury risk of 74% has been reported in youth players with a history of at least one previous injury [70]. Likewise, a ninefold increase in knee injuries was reported in senior players who had previously suffered an ACL injury [66]. Furthermore, the risk of sustaining a new injury increased with the number of previously reported injuries [62]. Whilst previous injury history appears important in determining future risk, arguably modifiable risk factors are of greater interest as action can be taken to reduce their impact with the aim of minimising the number of initial injuries.

Identified modifiable intrinsic risk factors in players include reduced knee alignment, i.e. increased dynamic valgus and high abduction loads [71, 72] as well as decreased knee and hip flexion angles [73] during landing. In addition, reduced lower body strength [71], a lower hamstring/quadriceps (H/Q) ratio during concentric action [74], generalised joint hypermobility (laxity) [74, 75] and specifically knee hyperextension [74] have also been shown to increase the risk of lower-limb injuries. Other risk factors, both modifiable and non-modifiable, include: increased age [64, 67, 68, 70, 75, 76], increased body mass index (BMI) [61, 66, 76], increased height [61], familial disposition [64], playing position [61, 71] (with forwards and defenders at greater risk), high training/match exposure [71, 74], single sport participation [71], time of the season [67, 77, 78], increased playing history [62], increased competitive level [70], hormonal fluctuations [79, 80] and psychological factors such as trait anxiety and negative life event stress [77].

### Prevention Strategies and Risk Management

As previous injury is considered the largest risk factor for sustaining an injury [81], it is reasonable to suggest that complete recovery is crucial to help prevent re-injury [82]. A specific rehabilitation programme will customarily address the injured site and focus on alterations in strength, proprioception and kinematics which may have occurred as a result of the injury or the time loss from training/match-play [83]. It is important to recognise that the framework of return to play milestones, directed by the medical physician and physical trainer, can be supported by the integration of sports psychology and sports nutrition [84]. Therefore, an interdisciplinary approach is recommended.

Exercise-based injury prevention programmes (IPPs) aim to improve whole-body biomechanics [81] and consist of a range of exercises focusing on physical components, including strength, balance, mobility, agility, plyometrics and running [81]. Some of the common IPPs are: FIFA 11 + [85], Prevent injury and Enhance Performance (PEP) [86], Knäkontroll [87] and Footy First [88]. A recent systematic review [81] identified that multiple-component IPP programmes can reduce overall injury rates (27%) and specifically ACL injury rates (45%). Such programmes were also found to be superior to single component programs in reducing the incidence of injury (38% and 22%, respectively). However, the effectiveness of IPPs may vary between cohorts; for example, the PEP programme significantly reduced ACL injury rate in youth players [86], yet despite lower injury rates, no significance was found in collegiate level players [89]. Finally, the beneficial effects of IPPs appear greater in females considered *high-risk* of injury as opposed to those classified as *low-risk* [90, 91].

Despite the effectiveness of IPPs, successful dissemination, implementation and adherence to such programmes remains a challenge [92]. Indeed, 83% of elite male soccer clubs failed to implement a specific hamstring injury prevention programme, despite 88% of clubs being aware of the programme’s effectiveness [93]. In female soccer players, the efficacy of IPPs is compromised by poor adherence [92, 94]. Overall injury risk is reduced (72%) in players with high adherence compared to players with moderate adherence [92]. The role of the coach is crucial, as positive coach attitudes towards IPPs correlate with high compliance and lower injury risk [94].

## Health

### Menstrual Cycle and Performance

**Fig. 1:**
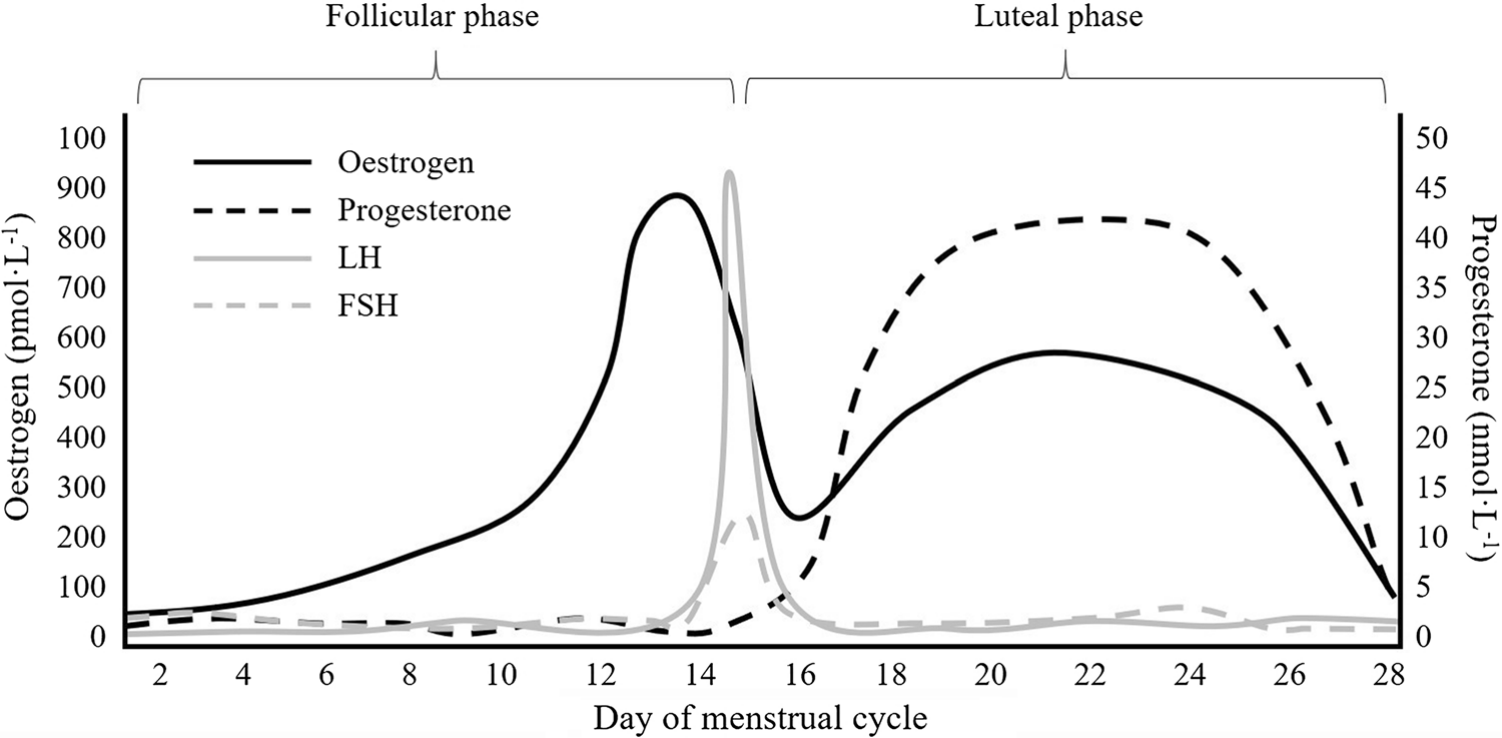
Graphical representation of oestrogen, progesterone, luteinising hormone (LH) and follicle-stimulating hormone (FSH) concentrations during a “typical” menstrual cycle

The menstrual cycle results in large variations in the concentration of reproductive hormones (Fig. 1). These variations could hypothetically influence soccer performance via direct effects of hormones (e.g. oestrogen or progesterone) on physiological function, or due to side effects of the menstrual cycle such as pre-menstrual syndrome or dysmenorrhea (e.g. cramps, headaches and nausea). A recent meta-analysis showed that exercise performance may be trivially reduced during the early follicular phase, although there was large variation in results between studies and much of the research was rated as low quality [95]. In particular, inter- and intra-participant variability in menstrual cycle characteristics (e.g. phase length and hormonal profile) can significantly affect study interpretation in the absence of rigid methodological control (for review, see [96]). Limited research has been conducted in soccer specifically; however, Julian et al. (2020) showed that very high-intensity running distance during matches over a 4-month period was significantly greater in the luteal phase (6.64 ± 2.72 m·min^−1^) compared to the follicular phase (5.90 ± 2.16 m·min^−1^), albeit with large measurement variance across matches [97]. Other studies in soccer players have shown no effect of menstrual cycle phase on power, repeated sprint ability and endurance [98, 99]. However, as with most research in this area, participants were studied at a group level to assess if there were differences in mean responses between phases, whereas the effects of the menstrual cycle are highly individualised. This is supported by 77% of elite athletes reporting negative side effects of the menstrual cycle with 24 distinct symptoms varying in intensity, duration and timing between individuals [100] and more than half of athletes perceiving their performance to be affected at certain points during their cycle [101, 102]. Given the individuality of symptoms and lack of coherence in the existing research, it is currently too early to provide any general guidelines in relation to the potential impact of the menstrual cycle on soccer performance. However, it is recommended that players/practitioners track menstrual cycles and symptoms to improve awareness of any phase-related effects on individual performance with a view to consideration of management strategies [100, 102].

### Menstrual Cycle and Injury

Women are 2–6 times more likely to have an ACL injury [103] compared to men (see Sect. 5). Several studies have shown that ACL injuries occur more commonly prior to ovulation when oestrogen concentrations are highest [104–106], which is associated with increased ligament laxity [107]. It should be noted, however, that limited research exists in team sports and the quality of existing studies is often poor, so no meaningful practical advice can be given in relation to the menstrual cycle and injury risk at present.

### Menstrual Cycle and Responses to Training

A recent meta-analysis showed the greatest levels of delayed-onset muscle soreness and strength loss following exercise occur during the early follicular phase when oestrogen concentrations are lowest [108]. Whilst this was based on limited data, practitioners could be mindful that some players may a need greater recovery duration (or at least focus) in the early follicular phase. Oestrogen has been proposed to increase the anabolic response to exercise, while progesterone has been associated with catabolic properties [107, 109]. Therefore, it has been hypothesised that the follicular phase is more favourable for exercise adaptations compared to the luteal phase, due to the greater oestrogen to progesterone ratio, especially in the late follicular phase (Fig. 1). Greater responses to training have been observed in studies when higher frequency/volume training was performed in the follicular phase compared to the luteal phase [110, 111], or compared to when training was evenly distributed across the cycle [111, 112]. However, not all studies have shown differences in training response between phases [113]. Phase-based training is a promising area to maximise training responses in soccer players. However, further research is required before implementation, due to the logistical challenges of personalising training loads in squads with asynchronous menstrual cycles.

### Hormonal Contraceptives

Approximately, half of all elite female athletes use hormonal contraceptives [100], with combined oral contraceptives (OC) being the most common (70%). As with the menstrual cycle, the reported symptoms with hormonal contraceptive use are highly individual, emphasising the need to work with players on a case-by-case basis. A meta-analysis showed that phase of the combined OC cycle (i.e. pill consumption or pill withdrawal) is unlikely to affect exercise performance [114]. Exercise performance was slightly inferior in OC users compared to naturally menstruating women, but the difference was trivial [114]. To date, no research has explored the impact of progestin-only contraceptives (e.g*.* coil, implant, injection and progestin-only OC) on exercise performance. This is despite progestin-only contraceptives accounting for ~ 30% of hormonal contraceptive use in elite athletes [100]. Furthermore, few studies have assessed the effects of hormonal contraceptives on training adaptations. To date no differences in responses to training for endurance performance or muscle strength have been found between OC users and non-users [115–117]. The type of OC, however, has been shown to affect responses, with antiandrogenic (i.e*.* opposing actions of testosterone) [118] and lower dose oestrogen (20 µg ethinyl oestradiol [115]) OC use resulting in poorer strength outcomes compared to OCs containing 30–35 µg ethinyl oestradiol and 2nd- and 3rd-generation progestins [100]. Further research is required before hormonal contraceptives can be differentially considered for performance benefits and/or training adaptations. It is also important to consider that there are ethical considerations for prescribing steroids for intended performance enhancement and that hormonal contraceptives are a prescription medication to be dispensed by medical practitioners only [100]. Combined OC use has also been associated with reduced bone mineral density in comparison to non-users, particularly in adolescent and young women [119, 120]. This could be an additional consideration for medical practitioners, although conversely a case–control study showed that fracture risk was lower in contraceptive users compared to non-users [121].

### Energy Availability

Energy availability (EA) is the amount of dietary energy available for normal physiological function after accounting for energy expended in exercise, expressed in proportion to lean body mass (LBM) [122]. Low EA has been associated with negative health and performance consequences such as menstrual dysfunction, osteoporosis and increased injury risk [123–126]. Low EA can be a result of intentional dietary restriction and/or increased energy expenditure in exercise to reduce body mass, as occurs with disordered eating/eating disorders and dieting, or an inadvertent failure to match energy intake to energetic demands [127]. Soccer players score lower than non-athlete controls on scores of disordered eating, and the proportion of self-reported eating disorders (5.9%) is lower than other sports and controls [128]. However, 8.0–19.3% of elite soccer players report menstrual dysfunction [129–131], a symptom of low EA. Prospective observations of EA show that 23.0–33.3% of top league players had low EA (< 30 kcal·kg LBM·day^−1^) during the in-season period [129, 132] and 26.3% had low EA during pre-season [132]. Within a typical training week, match day and heavy training (two exercise sessions) days result in a greater proportion of players with lower EA compared to rest or light (single session) training days [129]. This suggests that opportunities for energy intake should be maximised on match and heavy training days to prevent a recurring pattern of sub-optimal EA. This was a consequence of dietary energy intake not matching energetic demands [129]. Collectively, it appears that the majority of incidence of low EA occurred when energy intake was not adjusted to the energetic demands of training or match-play. Focusing on adequate energy intakes at lunch and evening meals could offer a useful starting point for support staff as previous work reports a link between low EA during the mid-season and relatively low dietary intakes during these meal times [132]. However, energy intake at other time points should not be neglected, as large within-day energy deficiencies can negatively impact metabolism independent of daily energy availability [133]. Teams might benefit from monitoring EA at times when sub-optimal EA is more likely (e.g. pre-season and in-season), although accurate measurement of EA is associated with several methodological challenges (for review, see [134]). In addition, practitioners should create environments that offer ample opportunities for food intake to support increased energy needs, coupled with player education on the negative consequences of low EA. Furthermore, prospective monitoring of menstrual cycle length should be undertaken as extended menstrual cycles can be a sign of low EA. It should be noted, however, that hormonal contraceptives may mask any effects of low EA on the regulation of menstruation. Thus, the presence of regular menstruation in hormonal contraceptive users is not indicative of EA status.

## Nutritional Strategies for Female Soccer Players

Nutrition plays an important role in soccer performance [135, 136]. What players eat can influence performance, recovery, growth, maturation, illness risk and general health. While a comprehensive review of the dietary requirements is beyond the scope of this review, this section will briefly review the main nutritional considerations for female soccer players.

To optimise health and performance, players should ensure they consume a healthy well-balanced diet, as well as enough energy (e.g. kilocalories (kcal)/kilojoules (kJ)) to cover their training and match demands. One method to gauge players’ energy needs is to quantify total daily energy expenditure. Although the number of studies examining total energy expenditure in female soccer players is limited, it has been found that this fluctuate over the course of a weekly micro-cycle [137]. Furthermore, daily exercise energy expenditure (EEE), reported by Moss et al. [129], was found to be negligible on rest days (~ 15 kcal kcal·day^−1^), and as expected, higher on match days (~ 881 kcal·day^−1^) and heavy training days (~ 786 kcal·day^−1^). Combined with an average resting metabolic rate of 1510 kcal·day^−1^, as measured by indirect calorimetry [129], these data suggest players' total daily expenditures are at least ~ 2400 kcal·day^−1^ on match days. The aforementioned studies used written logs, accelerometers and global positioning system units to measure total and EEE. The validity of using such methods has been questioned [138, 139]. To date, the use of doubly labelled water, the ‘gold standard’ method of calculating total daily energy expenditure, used in studies on male players is yet to be completed in females [139, 140]. Overall, the limited data available prevent formulation of generic energy intake guidelines for female soccer players. Instead, adequate EA should be encouraged for all players and any specific recommendations based on individual assessments.

Specific nutrition recommendations can be made in the days and morning before training and matches. The primary objective is to ingest adequate carbohydrate to ensure that glycogen availability does not limit the performance [141]. The amount of carbohydrate should be periodized and dictated by the anticipated duration and intensity of the activity [136]. Current recommendations suggest that 3–5 g·kg^−1^ of player’s body mass (BM)·day^−1^ is sufficient to fuel short-duration (< 1 h) skill-based sessions, but 6–10 g·kg^−1^ BM·day^−1^ is needed to fuel longer duration (1–3 h) high-intensity activities including matches [136, 142]. Observational studies suggest players typically consume ~ 3.3 to 5.2 g·kg^−1^ BM·day^−1^ of carbohydrates (reviewed in Dobrowolski et al., [143]). Whether carbohydrate intakes at the lower end of this spectrum compromise glycogen availability and performance is unknown. Female players have a lower net muscle glycogen utilisation but also a lower capacity for glycogen storage compared to males [144, 145]. Thus, intervention-based studies utilising incremental carbohydrate fuelling strategies are warranted in female soccer players.

Carbohydrate ingestion should also be prioritised following a match or training to replenish glycogen stores and restore subsequent endurance capacity [146, 147]. While moderate carbohydrate intakes are likely sufficient to restore glycogen concentrations after short-duration or low-intensity training sessions (3–7 g·kg^−1^ BM·day^−1^), higher intakes may be required for several days following matches (6–10 g·kg^−1^ BM·day^−1^). Indeed, a study in male soccer players showed that even with high carbohydrate intakes (9.5 g·kg^−1^ BM·day^−1^), glycogen restoration may take 48–72 h following matches owing to the presence of muscle damage [148]. Although females may experience less muscle damage than males [149], it would still be prudent to recommend high carbohydrate intakes in the days following matches to ensure glycogen availability is not compromised for subsequent training or matches.

Dietary protein stimulates muscle protein synthesis (MPS) and is important for the remodelling of muscle and connective tissues following exercise [150]. Soccer players are recommended to consume 0.25–0.40 g·kg^−1^ BM·day^−1^ of leucine-rich protein (e.g. whey, casein, dairy and egg) immediately following training or competition and every 2–4 h thereafter (equivalent to 1.2–1.7 g·kg^−1^ BM·day^−1^) to maximise MPS and recovery [151–153]. Dietary intake data suggest soccer players consume sufficient total daily protein (≥ 1.2 g·kg^−1^ BM·day^−1^; [129, 154–156] but little information is available for elite players or how these intakes are distributed. In males, high protein intakes (1.5 g·kg^−1^ BM·day^−1^) have been shown to attenuate symptoms of exercise-induced muscle damage (e.g. creatine kinase efflux and muscle soreness) following a match [157]. Although not soccer specific, a study in female dancers showed that higher protein intakes (1.8 vs. 1.3 g·kg^−1^ BM·day^−1^) accelerated the recovery of muscle function following intermittent sprint exercise [158].

During exercise, dehydration exceeding a 2% loss in body mass can impair aerobic capacity and cognitive function [159, 160]. A recent study in soccer players suggested that prior to matches and training ~ 47% of players were hypohydrated (as measured by urine specific gravity) [161], suggesting many players are not adequately re-hydrating between sessions. Consuming 5–10 ml·kg^−1^ BM of fluid in the 2–4 h prior to training or matches should ensure players are euhydrated once the activity starts [142]. During exercise, players should ingest enough fluids to avoid body mass losses of ≥ 2%, especially in hot environmental conditions [142]. Studies in female soccer players suggest body mass losses do not typically exceed 2% after 90 min of soccer-specific training, at least in cool temperatures, partly owing to the fact they have lower sweat rates than males [162, 163]. Thus, in most scenarios, ensuring players are hydrated prior to matches or training will be adequate. However, in hot temperatures, the risk and impact of hypohydration increases, and thus, there may be a greater need for individual hydration strategies that also consider electrolyte losses (especially sodium). In such scenarios, daily monitoring of body mass changes might help identify hypohydrated players.

Ergogenic aids including caffeine, dietary nitrate and creatine have been shown to enhance several aspects of soccer performance [164–166]. More specifically, improvements in intermittent running performance have been found following ingestion of both caffeine [167] and creatine [168, 169] in female soccer players. There is limited research, irrespective of sport, that examines the efficacy of anti-inflammatory supplements, such as phytochemical-rich cherry juice and curcumin, purported to accelerate recovery [170–172]. To date, more research is needed to determine whether pre-exercise ergogenic aids and recovery supplements, shown to benefit male soccer players, elicit similar effects in females.

## Summary and Conclusion

The six topics discussed in this review (physical demands, talent identification, body composition, injury risk and prevention, health and nutrition) are of pertinent interest to researchers and practitioners working in female soccer. We have attempted to summarise current best practices in women’s soccer, which may help to inform personnel working in professional soccer to establish sport science support, but also identify knowledge gaps and make suggestions for future research. More specifically, from a physical demands perspective, future research should use technology (MEMS) to more accurately determine the physiological cost of soccer-specific movements of female players. Previous studies provide a foundation to inform talent identification in female soccer; however, future research should now explore the utility of other contextually relevant methods (e.g. small and sided games) for identifying young talented soccer players. There is no consensus on the ideal body composition for female players and due to the sensitivity of regular assessment, it is recommended that organisations develop a body composition assessment framework for female soccer players. Exercise-based injury prevention programmes in female soccer are compromised by poor adherence; however, behaviour change interventions, coupled with positive reinforcement from support staff, may improve adherence rates. There are limited data on the influence of the menstrual cycle on performance, injury and training adaptations, and thus, this is a fruitful area of opportunity for future research. Preventing low EA is of paramount importance for the health and well-being of female soccer players. We recommend that practitioners educate players on the consequences of low EA and create environments that offer ample opportunities for food intake to support increased energy needs. Finally, there is a lack of sports nutrition research conducted in female players. Future studies are needed to confirm if the benefits of specific dietary patterns (e.g. high carbohydrate intakes) or ergogenic aids (e.g. dietary nitrates) translate to female players. It is important to recognise the barriers that may be experienced when conducting research with professional players within a professional club environment. In response, Table 7 aims to identify such barriers and offer potential approaches, with the aim of maximising the likelihood of a successful research partnership.

**Table 7:** Potential barriers to and suggested approaches for research in professional female soccer clubs

Barrier to research	Suggested approach
Coach resistance (inexperience)	Educate coach first to encourage buy-in and support
Navigating the hierarchical structures within the club	Initiate discussions with key leadership stakeholders within the club at the early stage of research development, and allow time to build relationships and trust
Avoidance of an academic-led research agenda/no research agenda within the club	Perform a scoping exercise with the coach, sport scientists and players and create a strong dialogue within the club to identify “real world” issues that the research/academic can address, and support clubs to set up a research strategy
Player resistance	Target key/ influential players (i.e. club captain) to help ensure team buy-in
Confidentiality	Allow time to build trust with support staff and players
Lack of staff/staff time	Partner with academic institution
Interruption to player training and time	Ensure rapid data turn around and provide meaningful feedback to players and support staff

In conclusion, the demand for knowledge in female soccer has outpaced the empirical evidence base. Thus, high-quality investigations, specific to female soccer, are needed to inform recommendations and improve our understanding of how best to support the health and performance of female players.
